# Aim high energy conversion efficiency in triboelectric nanogenerators

**DOI:** 10.1080/14686996.2020.1800366

**Published:** 2020-09-28

**Authors:** Hong-Joon Yoon, Sung Soo Kwak, Seong Min Kim, Sang-Woo Kim

**Affiliations:** School of Advanced Materials Science and Engineering, Sungkyunkwan University (SKKU), Suwon Gyeonggi-do, Republic of Korea

**Keywords:** Mechanical energy harvesting technology, triboelectric nanogenerators, high polarity, high energy conversion efficiency, 206 Energy conversion, transport, storage, recovery

## Abstract

Triboelectric nanogenerators (TENGs) that enable the conversion of a given mechanical energy into electrical energy at high efficiency have been very important in practice. Since the given mechanical energy is involuntarily converted to secondary energy sources (light, heat, and sound during triboelectrification), the significant amount of energy being converted is lost. Various studies have thus been continuously carried out to overcome this issue. Since the first TENGs found in 2012, various developments in TENGs have been made: (1) the mechanical-electrical energy conversion characteristics of potential organic/inorganic material groups have been introduced, (2) the integration into the device structure considering the diversity of mechanical energy, and (3) user friendly and industrial application platforms have been aggressively studied. Despite the remarkable progress and improvement of TENGs, their mechanical-electrical conversion efficiency is still quite low. We therefore need to discover and develop materials that can be converted to improve efficiency. Here, we outline the recent progress made in a group of high polarity triboelectric materials that exploit surface charge density and charge transfer properties. We also review the recent boosting powering TENGs. The aim of this work is to provide insight into the future direction and strategies for highly enhanced powering TENGs through material research.

## Graphical Abstract

**Figure:**
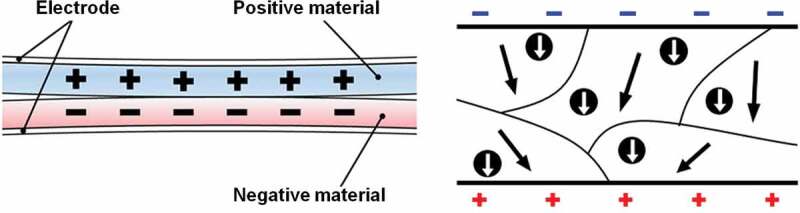

## 1. Introduction

As the Fourth Industrial Revolution comes, emerging technology breakthroughs in a number of fields such as artificial intelligence, quantum computing, biotechnology [1], The Internet of Things (IoT), and autonomous vehicles become embedded within societies and even human body. Especially, human-machine interaction or so-called multimodal interaction provides the users with multiple modes of interacting with a smart system, which inevitably results in demanding energy consumption [2]. Therefore, many forms of rechargeable battery systems are emerging. However, the cost and labor of charging are increasing, and even the battery exchange during the emergency is technically impossible, in contrast to technological advances. To solve this problem, alternative energy technology that can convert the energy of the surrounding into usable energy for directly charging the battery and developing self-powered platform has appeared [3–7], and the application of the technology to convert the mechanical energy triggered from the interaction with the environment into the electrical energy has been highlighted [8].

Among alternative mechanical energy technologies, triboelectric nanogenerators (TENGs) have been developed as rising energy harvesting devices were first introduced by Prof. Zhong Lin Wang’s group. Recently, research in TENGs has been prominent due to its relatively high output power [9]. The TENGs based on contact electrification and electrostatic induction physics have been developed as a self-powered platform and powering devices driven by mechanical contact or friction triggered by object movement. Like all energy conversion technologies, TENGs are evaluated by an efficiency that determines possible operate-able application range. The efficiency of TENGs can be affected by two stages; an initial stage in conversion (ISC) is regarding a process that kinetically triggered materials get into contact with opposite materials is followed by instant electrical charge induction along electrodes. The final stage in conversion (FSC) is regarding a circuit efficiency that induced electricity is how efficiently converted to store/use in applications. One of the important factors to influence the ISC can be quantified as the ratio of the amount of induced surface charges to the polarization of materials, and another as the efficiency of the circuit part can be referred to as the FSC. Since the FSC has been sufficiently developed for a long time, more than 90% can be technically realized [10]. However, based on the laws of conservation of energy, given mechanical energy is converted to other energy sources (e.g., light and sound on contact area) rather than electrical energy, resulting in such a low ISC. It determines the energy output spectrum of the TENGs like many alternative energy technologies [11].

There have been numerous efforts to extend this range of energy output since 2012. In any case, the structure of the TENGs is designed according to four representative driving modes; (i) single electrode mode, (ii) contact mode, (iii) sliding mode, and (iv) free-standing rotation mode [12–15]. Depending on the modes, many structural and related application studies have been actively conducted. TENGs have been explored and studied based on a variety of existing materials in these four modes since all materials in the universe exhibit distinct electrification characteristics upon contact with other substances [16]. In addition to device and application studies, materials exhibiting the highest output and conversion efficiency from a material standpoint were expressed when TENGs were fabricated based on those with strong polarity properties [17].

We begin this review with the current understanding of the implementation of newly developed high polarity properties in the active materials in TENGs. Based on previous studies, it is concluded that the chain length manipulation of polymers and embedding high dielectric materials inside the polymer matrix are influential on the level of polarization of materials. More importantly, enhanced polarization of materials is closely related to the magnitude of induced surface charge density. Now that considering the working mechanism of the TENGs, the increase of surface charge density will increase the possibility of charge exchange between surfaces upon contact/separation, which is resulting in enhanced output performance of the TENGs. In addition, we consider that enhanced surface charge density can chemically dope the polymer electrolyte with various ion pairs. In the final section, we propose a future direction with strategies for developing high ISC triboelectric materials toward extending the energy output spectrum as alternative energy technology.

## 2. Understanding of polarity on TENGs

**Figure 1.**
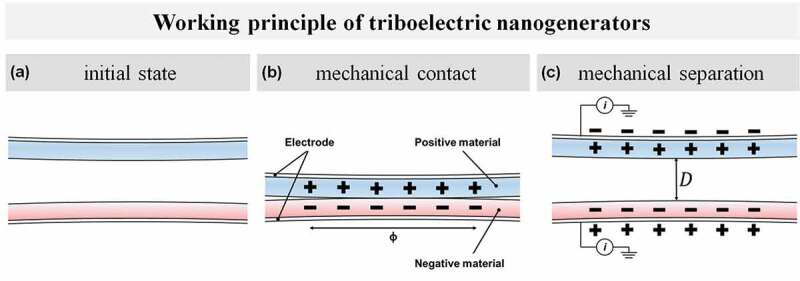
Schematics of working principle of TENGs (a) Initial state: two different tribopositive/tribonegative materials present with a certain distance, (b) Mechanical contact: mechanically triggered objects make contact with surface area, Ф. Transient polarization at the surface level induced. (c) Mechanical separation: objects are separated with distance, *D*. Current flows in order to balance each temporarily unbalanced electrically polarized surface.

Forming charges on a material surface upon mechanical contact and separation is the initiator of triboelectricity. Any kinetically triggered objects get into contact will exchange charges at the surface level depending on a few parameters including structural parameters such as dielectric thickness and separation distance, and material parameters such as work function, surface potential, electron affinity, and internal polarity [18,19]. As described in Figure 1, a charge exchange between materials surface Ф will induce a transient charge transfer on the interface with equal and opposite polarity (Figure 1(b)) after getting contact. This state is temporarily equilibrated, which is inclined to be maintained. As the materials separate, the formed interface polarization tends to break away. Under the premise of insulators, where charges (or electrons) cannot freely move, the amount of exchange charges remain on each surface after materials are mechanically separated (Figure 1(c)). In order to overcome this oppositely biased charge characteristic on surfaces and to become electrical equilibrium, the free electrons flow along the external circuit connected to each material. Described whole phenomena are representing the fundamental principle of TENGs and the conversion of given mechanical energy into a valued amount of electricity. The output performance of TENGs can be determined by the amount of charges exchange, in other words, the level of interfacial polarization.

Triboelectricity (called static electricity), which has been regarded only as a phenomenon in nature for a long period of time, convert into electrical energy as a power source since the first TENGs had introduced in 2012, and its related research in diverse aspects have been conducted. Any materials can be used as triboelectric materials as long as it accepts/donates electrons while rubbing with opposite materials, but the difference in the level of polarity between contacted materials is believed to be a key feature to develop high performing TENGs [20].

Regarding the origin of TENGs, mechanical energy conversion tendency is quite dependent on the mechanical characteristics of the triggered objects (frequency, amplitude, acceleration, orientation), which has been investigated not only theoretically but also experimentally [21]. On the basis of a few driving TENGs modes, there has been much research on materials with high robustness and polarization property. In general, charge exchange on the surface between materials is mainly governed by the work function difference. Besides, an electron affinity is also serving as an important parameter regarding the performance of TENG because fluorinated polymers have been observed to provide high charge exchange property. Therefore, the main subject of the TENGs materials study has focused on F atom-based polymer materials.

## 3. Current research and impact of polarity on TENGs

**Figure 2.**
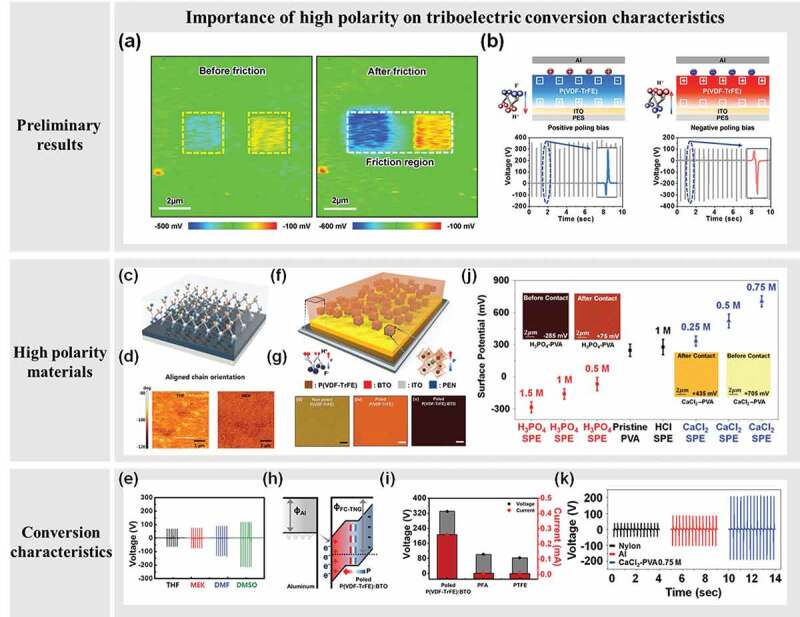
Schematics showing the importance of polarity for the triboelectric conversion characteristics. (a) Induced polarization on ferroelectrics characterized by KPFM, and (b) TENGs tuned as electropositive/electronegative ferroelectric polarity and electrical output characteristics. Reproduced by permission from [22], copyright [2016, Wiley-VCH] (c) Well aligned polymer chain based TENGs, and (d) the impact on surface charge density characteristics depending on the chain length. (e) Conversion characteristics trend depending on the polarity of polymer. Reproduced by permission from [23], copyright [2017, Wiley-VCH] (f) Nanocomposite structure capable of excessive polarity, and (g) controlled surface charge density through the level of polarity with its (h) working principle and (i) superior conversion characteristics beyond current TENGs materials. Reproduced by permission from [24], copyright [2017, Wiley-VCH] (j) Electrolyte based TENGs materials controlled triboelectric charge, and (k) the electrical conversion characteristics. Reproduced by permission from [25], copyright [2017, Wiley-VCH]

First, it was necessary to understand the influence on the TENGs output performance according to the level of ferroelectric polarity and its characteristics whether tribonegative or tribopositive. When it comes to analyzing the effect of ferroelectric polarity on the TENGs, one of ferroelectric polymer, poly(vinylidene fluoride-trifluoroethylene) [P(VDF-TrFE)] is a suitable candidate to control its ferroelectric polarity as modulating outermost elements [F or hydrogen (H)] through a certain level of electric field application (poling) [22]. As shown in Figure 2, the ferroelectric polarity of the surface was confirmed by Kelvin probe force microscopy (KPFM). By applying a positive potential internally, the outermost atoms were dominated by F atoms, while the outermost atoms were dominated by H atoms when a negative potential was applied. In each case, a region with a positive potential has an electropositive characteristic, whereas a region with a negative potential has an electronegative one. We not only verified that the controlled polarity affects the surface potential at the atomic level but also confirmed its electrical properties of the device at centimeter scale. Consequently, the mechanical-electrical conversion characteristics can be controlled and its efficiency can be enhanced by modulating the polarity of the TENGs materials.

It was verified that the mechanical-electrical energy conversion characteristics are affected by the presence and direction of the polarity of the material and its level. This polarity can also be improved by its order and uniformity. Considering the characteristics of the polymer materials, the chain length can be controlled through various means during synthesis, and that is likely to determine the crystallinity [23]. A polymer having high ordered polarity means that the degree of domination by the same outermost element of a given surface is increased, resulting in an improved electronegativity or electropositivity. We have understood the surface chemistry characteristics of polymers due to the chain lengths depending on the type of solvent in the polymer synthesis process. Taking advantage of this strategy, we understood high conversion characteristics of TENGs with given high electropositive/electronegative polymers. When it comes to the conversion characteristics, it is necessary to analyze the surface charge density of the present material before and after the contact with the counterpart materials. Through the contact potential difference (CPD) analysis strategy, the controlled electropositive (tribopositive)/electronegative (tribonegative) characteristics were studied and verified at the atomic level. In addition, it was confirmed experimentally by fabricating a macro-scale TENGs device with a centimeter size, and its current output characteristic was improved by about 75% by controlling the polarity of the solvent (Figure 2(c-e)). We verified the effect of polymer chain length on the degree of orientation uniformity of polarity, and as a result, the quantitative correlation between the energy conversion characteristics and the polarity of the polymer.

In addition, various research efforts have been made to realize excess polarity characteristics in the limiting polarity characteristic of conventional inherent polymers. There has also been a report that an excess polarity characteristic can be achieved by injecting ions from an external system (e.g., air) into a polymer material. We have introduced a technique to optimize the highly excessive polarity conditions according to a certain fraction of nanoparticles through the polymer matrix/nanoparticle composite structure design and thus achieved an improved mechanical-electrical conversion property of TENGs device [24]. As embedding ferroelectric/high dielectric controllable nanoparticles, BaTiO_3_ (BTO) within the P(VDF-TrFE) matrix, randomly aligned polarity of the BTO nanoparticles will invigorate the electronegativity of a given ferroelectric matrix polymer once the electric field is applied, resulting in an excess polarity composite system (Figure 2(f)). The highly excessive polarity of this composite system was analyzed through the KPFM tool before and after mechanical contact with the counterpart material to understand the enhanced polarity of the material (Figure 2(g-h)). When it comes to power conversion, the improved tribonegativity on the surface due to this excess polarity resulted in a conversion efficiency of about 150 times higher than that of the conventional F-based polymer-based TENGs (Figure 2(i)). In this regard, any materials, serving as a matrix, can have improved polarity by taking advantage of the composite structure.

In previous studies, excessive polarity has been achieved through physically embedding techniques through composite structures, but studies have also been conducted to chemically control excessive polarity [25]. Through a simple chemical doping process, the intrinsic polarity of polyvinylalcohol (PVA), a well-known electrolyte substance, has been exceeded. We have realized the highly excessive polarity characteristics through the combination of various ion pairs and concentration control to modulate the polarization of neutral PVA material, and have developed the relatively tribonegative/tribopositive characteristic control technology. These controlled polarity properties were verified by KPFM analysis and they acted as active materials of TENGs by depositing them on the substrate (Figure 2(j)). Therefore, we experimentally confirmed the mechanical-electrical conversion efficiency was improved even at the macro-level scale (Figure 2(k)). This approach is groundbreaking to develop a system of excessive polarity by chemical doping technology and to be applicable to TENGs because of the advantage that it is possible to introduce broad material groups through surface chemistry technology. This study opens the possibility of the unexplored materials to become active TENGs materials that have not yet been discovered.

## 4. Outlook and perspective

**Figure 3.**
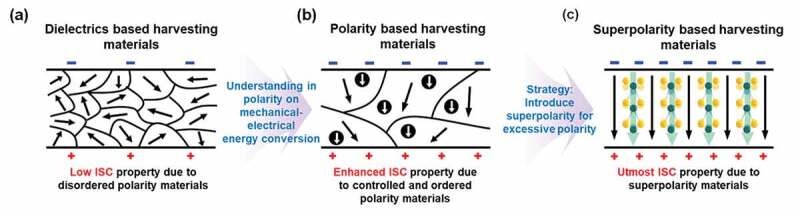
Schematics of enhancing the initial stage in conversion (ISC) of TENGs. (a) Low ISC: disordered polarity based TENGs, (b) Enhanced ISC: controlled and ordered polarity based TENGs, (c) Utmost ISC: superpolarity based TENGs.

We have entered the stage of ‘maturity investigation’ from an early stage of that as to increase tribopositive/tribonegative properties due to excessive polarity and thereby enhance mechanical-electrical conversion efficiency. The main variables that affect the polarity are countless. We need to proceed with a screening process in which a group of substances will be examined at a future ‘intensive investigation’ stage. Regarding fundamental physical chemistry, it would be necessary to understand, for example, what elements, molecular bonds, and functional groups affect the magnitude of polarity through atomic-level density functional theory and molecular-level molecular dynamics calculation. Based on this, it is necessary to not only quantitatively but also qualitatively analyze the polarity ambiguity of certain materials. Through this, we will be able to select the material framework of interest for the intensive investigation, which will enable the development of superpolarity materials through strategic synthesis. This successful superpolarity synthesis and maximization of the tribopositive/tribonegative characteristics can increase the ISC (Figure 3). It will bring synergies on current TENGs research, which has been somehow at the saturation investigation stage thus raise the level of the TENGs field. Through superpolarity-based TENGs, it might be possible to make a step toward the development of various self-powering smart systems (robotics, IoT related sensor platforms, consumer electronics, and portable electronics), and a power bank in various places. As a result, it will become an important research field that occupies a large part of the future promising mechanical energy-based alternative energy field. It is expected to extend the energy output spectrum that other alternative energy fields cannot cover, and help to overcome the limitation of the energy industry leaning on existing fossil fuels.
